# Prevalence of the Prescription of Potentially Interacting Drugs

**DOI:** 10.1371/journal.pone.0078827

**Published:** 2013-10-11

**Authors:** Elena Tragni, Manuela Casula, Vasco Pieri, Giampiero Favato, Alberico Marcobelli, Maria Giovanna Trotta, Alberico Luigi Catapano

**Affiliations:** 1 Epidemiology and Preventive Pharmacology Centre (SEFAP), University of Milan, Italy; 2 Institute of Leadership and Management in Health, Kingston University, Kingston Hill, Kingston upon Thames, Surrey, United Kingdom; 3 Information Flow and Economic Analyses, Regional Health Unit, Marche, Italy; 4 General and Hospital Practice and Drug Policies, Regional Health Unit, Basilicata, Italy; 5 IRCCS Multimedica, Sesto San Giovanni, Milan, Italy; University of Manitoba, Canada

## Abstract

The use of multiple medications is becoming more common, with a correspondingly increased risk of untoward effects and drug-related morbidity and mortality. We aimed at estimating the prevalence of prescription of relevant potentially interacting drugs and at evaluating possible predictors of potentially interacting drug exposure. We retrospectively analyzed data on prescriptions dispensed from January 2004 to August 2005 to individuals of two Italian regions with a population of almost 2.1 million individuals. We identified 27 pairs of potentially interacting drugs by examining clinical relevance, documentation, and volume of use in Italy. Subjects who received at least one prescription of both drugs were selected. *Co-prescribing* denotes “two prescriptions in the same day”, and *concomitant medication* “the prescription of two drugs with overlapping coverage”. A logistic regression analysis was conducted to examine the predictors of potential Drug-Drug Interaction (pDDIs). 957,553 subjects (45.3% of study population) were exposed to at least one of the drugs/classes of the 27 pairs. Overall, pDDIs occurred 2,465,819 times. The highest rates of concomitant prescription and of co-prescription were for *ACE inhibitors+NSAIDs* (6,253 and 4,621/100,000 plan participants). Considering concomitance, the male/female ratio was <1 in 17/27 pairs (from 0.31 for *NSAIDs-ASA+SSRI* to 0.74 for *omeprazole+clopidogrel*). The mean age was lowest for *methotrexate* pairs (+*omeprazole*, 59.9 years; +*NSAIDs-ASA*, 59.1 years) and highest for *digoxin+verapamil* (75.4 years). In 13/27 pairs, the mean ages were ≥70 years. On average, subjects involved in pDDIs received ≥10 drugs. The odds of exposure were more frequently higher for age ≥65 years, males, and those taking a large number of drugs. A substantial number of clinically important pDDIs were observed, particularly among warfarin users. Awareness of the most prevalent pDDIs could help practitioners in preventing concomitant use, resulting in a better quality of drug prescription and potentially avoiding unwanted side effects.

## Introduction

Quality assessment and improvement in health care is a major issue in many countries. Information on healthcare is in demand from policy makers, health-care professionals and the general public. With the majority of doctor-patient encounters in general practice resulting in a prescription for drug treatment, the quality of prescriptions is a critical issue as prescribing drugs has a major influence on patients’ well-being, and accounts for a substantial part of health care expenditure. 

Drugs are often used in combination to achieve a preferred therapeutic goal or to treat coexisting diseases. Because of the risk related to concomitant use of drugs, co-medication has become a general concern and an important concept in term of prescribing appropriateness. Some combinations may result in undesired pharmacodynamic or pharmacokinetic interactions, resulting in undertreatment or harmful effects [1]. The consequences of drug-drug interactions (DDIs) can range from no untoward effects at all, to drug-related mortality. Although DDIs are considered to be preventable, studies up to 11% of patients experience symptoms associated with DDIs [2], and DDIs are responsible for up to 2-3% of hospital admissions [3,4]. DDIs are associated with increased health care use [5,6]. In the United States, the economic burden of medication-related morbidity and mortality is as high as $177 billion [7].

Although DDIs are one of the most significant problems with drug prescribing [8], most physicians are not fully aware of all major and clinically important drug interactions [9,10], or underestimate the risk of the co-administration of multiple drugs [11]. Furthermore, the pharmacist rarely intervenes when it recognizes the presence of a potentially clinically important DDI [12,13]. Research using prescription databases can contribute to a better understanding of potential DDIs (pDDIs); however, only a few studies have examined clinically important DDIs in an outpatient setting, and even fewer have identified patients at risk [14,15]. 

The aim of this study was to estimate the prevalence of some contraindicated/major/moderate pDDIs in the population registered under the Regional Health Authority of Marche and Basilicata (central and southern Italy, respectively) during the period 2004-2005, and to evaluate the association of pDDI with available patients' characteristics, as age, gender and number of prescribed drugs.

## Methods

This observational, cross-sectional study was part of the ASSET (Age and Sex Standardised Estimates of Treatment) project [16], a pharmacoeconomic and pharmacoepidemiological study. This analysis focused on data from the Regional Health Departments of Basilicata (a southern Italian region with almost 600,000 inhabitants) and Marche (a central Italian region with almost 1.5 million inhabitants), with a population of slightly more than two million subjects (ASSET population), 1738 general practitioners (GPs) and 244 family paediatricians (FPs).

In Italy, retrospective studies using administrative prescription databases do not require Ethics Committee (EC) protocol approval or notification [17] therefore we did not request approval from the EC, nor consult with the EC to receive a formal written waiver .

### Data sources

The Basilicata and Marche Regional Health Departments collect prescription data from all Local Health Units of the regions on a monthly basis. These data are grouped in a regional database that can be linked to other administrative databases (e.g. with patients’ personal data) using a unique specific identification code. These prescriptions refer only to drugs covered by the Italian National Health Service that are prescribed by GPs and FPs and dispensed by community pharmacies. The regional prescription database includes a full account of product dispensed, dates of prescription and dispensation by community pharmacies, and the personal identification codes for each patient who receives a prescription. All prescriptions were classified according to the Anatomical Therapeutic Chemical (ATC) classification system, as recommended by the WHO [18], and were identified by their AIC (authorisation for marketing) number, which allowed us to determine the specific details and calculate the duration of each prescription (number of units and dosage). The demographic data for patients (sex and date of birth) were available from a regularly updated *ad hoc* regional database, which could be linked through patient identification keys.

All personal data (name and identification number) were replaced by a univocal numerical code, making both databases anonymous at source in strict compliance with the Italian Privacy Law (Decree 196, 30/06/2003). The study design (observational and retrospective in nature) meant that informed consent was not required from the subjects (Decree 196/03, art. 110).

### Choice of drug pairs

Potential DDIs were identified using the Micromedex® interaction database. In this system, all drug interactions are classified according to two parameters. **Clinical relevance** is the first, and takes into account potential clinical outcomes, and the type, quality, and relevance of supporting clinical data. The classifications for clinical relevance are: *Contraindicated* (the drugs are contraindicated for concurrent use), *Major* (the interaction may be life-threatening and/or require medical intervention to minimise or prevent serious adverse effects), *Moderate* (the interaction may result in exacerbation of the patient's condition and/or require an alteration in therapy), *Minor* (the interaction would have limited clinical effects; manifestations may include an increase in the frequency or severity of the side effects but generally would not require a major alteration in therapy), and *Unknown*. The second parameter is **pharmacological documentation**: the classifications in this case are *Excellent* (controlled studies have clearly established the existence of the interaction), *Good* (documentation strongly suggests the interaction exists, but well-controlled studies are lacking), *Fair* (available documentation is poor, but pharmacological considerations lead clinicians to suspect the interaction exists, or the documentation is good for a pharmacologically similar drug) and *Unknown*. 

The choice of drug pairs was made according to the following criteria:

- Contraindicated/Major/Moderate for clinical relevance and/or Excellent/Good for documentation;
- High position in the rankings of use in Italy, on the basis of OSMED 2004 (an annual national report on drug utilization and expenditures) [19] for at least one component of the pair.

The components in the pairs could be a drug class or a single molecule.

A total of 27 pairs were identified (Table **1** and Table **S1**), involving 144 drugs overall (17 single drugs and 8 drug classes). 

**Table 1:** List of the 27 pairs.

**1**	Simvastatin-Itraconazole	**15**	Simvastatin-Clarithromycin
**2**	Metformin-Fluoroquinolones	**16**	Betablockers-Verapamil
**3**	Omeprazole-Clopidogrel	**17**	Simvastatin-Verapamil
**4**	Warfarin-Amiodarone	**18**	Enalapril-Allopurinol
**5**	Warfarin-Moxifloxacin	**19**	Warfarin-(nsaids or asa)
**6**	Simvastatin-Amiodarone	**20**	Methotrexate-(nsaids or asa)
**7**	Warfarin-Simvastatin	**21**	Enalapril-asa
**8**	Digoxin-Verapamil	**22**	Enalapril-Metformin
**9**	Warfarin-ssris	**23**	Warfarin-Itraconazole
**10**	Verapamil-Atenolol	**24**	Warfarin-Levothyroxine
**11**	Heparines-(Nimesulide, Indomethacin, or ASA)	**25**	Simvastatin-Digoxin
**12**	Amiodarone-Antiarrythmics Ia	**26**	ace inhibitors-(nsaids or asa)
**13**	Methotrexate-Omeprazole	**27**	Ssris-(nsaids or asa)
**14**	Simvastatin-Gemfibrozil		

SSRIs: selective serotonin reuptake inhibitors, NSAIDs: nonsteroidal antiinflammatory drugs, ASA: acetylsalicylic acid, ACE: angiotensin converting enzyme

### Potential DDI assessment

**Figure 1:**
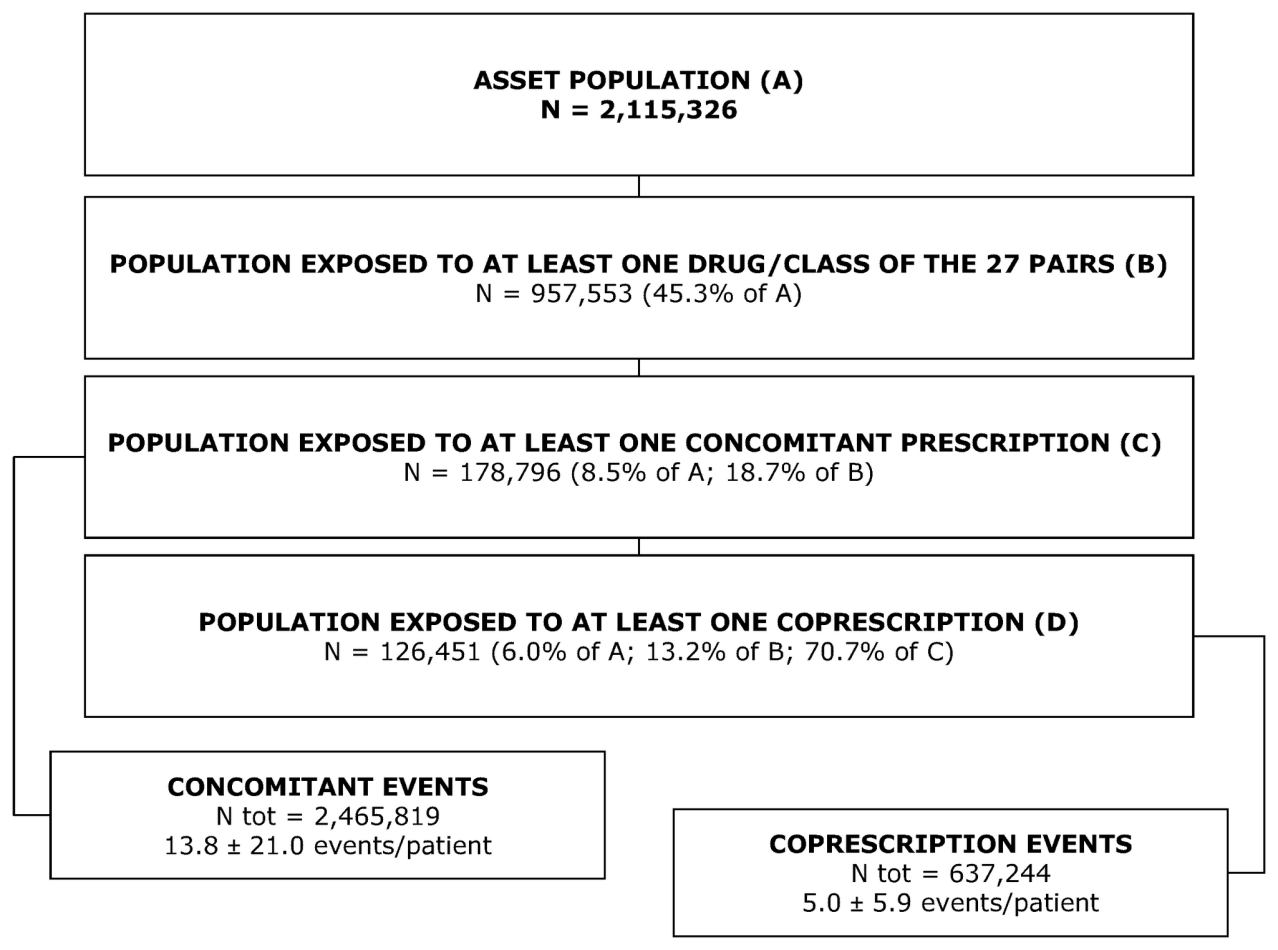
Number and proportion of patients involved in pDDIs.

To assess the frequency and distribution of pDDIs, all drug prescriptions registered from 1 January 2004 to 31 August 2005 were considered (Figure **1**). For each drug pair, people who received at least one prescription were selected to evaluate the presence of pDDIs and risk factors. We used defined daily doses (DDDs) from the ATC/DDD system [18] to construct a proxy measure for a day’s supply. We assumed a day’s supply for a particular prescription to be equal to the total amount of drug in the prescription divided by the DDD.

Several patterns of co-medication can be defined [20]. We investigated two patterns:

1 ***Co***-***prescribing*** is defined as “the joint prescription of more than one drug by the physician on the same day”.
2 ***Concomitantmedication*** is defined as "the prescription of two drugs with coverage partially overlapping in time, according to information from the pharmaceutical database".

Co-medication may also result from two drugs being available to the patient because they have been dispensed within a certain time period, and some pills are left over. This latter source of co-medication is strictly dependent on the patient's behaviour, while co-prescribing and concomitant medications denote co-medication resulting from the use of drugs as intended by medical doctors. 

### Statistical analysis

The unit of analysis was the individual subject. We enumerated individuals exposed to each of the 27 pDDI combinations, counting each individual only once for each combination, irrespective of the number of times he/she was exposed over the 20-month period. 

*Case-exposure rates* to the pDDIs were calculated as the number of people exposed to a pDDI divided by the number of individuals receiving one element of the pair. *Rates of exposure* to the pDDIs were calculated for each drug pair, and were expressed as the number of subjects with a pDDI per 100,000 plan participants (PP, i.e., the enrolled population). 

A multivariate logistic regression analysis in each drug cohort to determine the factors associated with pDDIs was performed. Exposure to a pDDI (Yes/No) was the dependent variable in the model. Patient characteristics incorporated in the model as independent variables included age (<50 [reference]; 50-64; 65-74; ≥75 years), gender (male [reference]; female), and the number of medications prescribed (<5 [reference]; 5-9; ≥10). 95% confidence intervals around each odds estimate were calculated. 

All analyses were performed using SPSS^®^ version 16.0.2 (SPSS Inc., IBM Company Headquarters, Chicago, Illinois, USA).

## Results

### General prevalence

The study population included 2,115,326 participants (3.6% of all Italians, population A) (Figure **1**). 

957,553 subjects (population B) (45.3% of the ASSET population) were exposed to at least one of the drugs/classes of the 27 pairs. Of this subpopulation, 18.7% had at least one overlapping prescription (*concomitant patients*, population C), and 13.2% received prescriptions of interacting drugs on the same day (*patients with co-prescription*, population D).

Overall, we counted a mean±SD of 13.8±21.0 concomitant events per patient, and 5.0±5.9 co-prescriptions per patient. In addition, 25% of patients received at least one concomitant pDDI in more than one pair, up to a maximum of 9 pairs. Considering co-prescriptions, the corresponding percentage decreased to 20%, up to 7 pairs.

### Distribution by sex, age and number of prescriptions

Table **2** (and Table **S2**) shows the main characteristics of the populations exposed to the 27 pairs of pDDIs. 

**Table 2:** Main characteristics of the populations, N (%).

	**POPULATION A (ASSET)**	**POPULATION B**	**POPULATION C**	**POPULATION D**
	N = 2,115,326	N = 957,553	N = 178,796	N = 126,451
**GENDER**				
**Male**	1,032,357	436,999	78,897	54,932
	(48.8)	(45.6)	(43.0)	(43.4)
**Female**	1,082,969	520,554	101,917	71,519
	(51.2)	(54.4)	(57.0)	(56.6)
**M/F**	0.95	0.84	0.75	0.77
**AGE CLASSES, years**				
**<50**	1,283,070	406,656	16,019	9,758
	(60.7)	(42.5)	(9.0)	(7.7)
**50-64**	375,404	225,370	41,856	28,299
	(17.8)	(23.5)	(23.4)	(22.4)
**65-74**	236,764	168,198	56,210	40,440
	(11.2)	(17.6)	(31.4)	(32.0)
**>=75**	220,088	157,329	64,711	47,954
	(10.4)	(16.4)	(36.2)	(37.9)
**PRESCRIBED DRUGS, n**				
**<5**	--	406,549	13,898	9,297
		(42.5)	(7.8)	(7.4)
**5-9**	--	350,488	66,723	46,210
		(36.6)	(37.3)	(36.5)
**>=10**	--	200,516	98,175	70,944
		(20.9)	(54.90)	(56.1)

Considering concomitant prescriptions, the male/female ratio was <1 in 17/27 pairs of drugs, with the smallest value for the *SSRIs +* (*NSAIDs or ASA*) pair (0.45) and the highest value for the *omeprazole + clopidogrel* pair (2.85). For co-prescription, the male/female ratio was <1 in 16/27 pairs of drugs, with the smallest and highest values observed for the same pairs as for concomitant prescriptions (0.43 and 3.06, respectively).

In the cohorts of concomitant prescription-exposed patients, the lowest mean ages (59.1±15.7; 60.0±15.4) were observed for *methotrexate* in both of its pairs (+ *omeprazole*; + [*NSAIDs or ASA*]). The mean age of the patients was ≥75 years for only one pair (*digoxin + verapamil*). In 13/27 cases the mean ages were ≥70 years. The same considerations could be made for patients with co-prescriptions. 

In both cohorts (of patients with concomitant or co-prescriptions), the mean number of prescribed drugs was ≥10.

### Patterns of co-medication

The rates of concomitant prescription (Table **S3**) were greatest for persons prescribed with *ACE inhibitors + NSAIDs* (6,253.4/100,000 PP), *SSRIs +* (*NSAIDs or ASA*) (1,589.7/100,000 PP), *heparines +* (*nimesulide, indomethacin, or acetylsalicylic acid*) (910.0/100,000 PP), enalapril + *ASA* (662.0/100,000 PP) and *metformin + fluoroquinolones* (428.6/100,000 PP). The same five pairs showed the highest rates of co-prescription events, with the same ranking. The ratio between the subjects with co-prescriptions and the subjects with concomitant prescriptions was 0.81 (the highest) for the *digoxin + verapamil* pair, and 0.24 (the lowest) for the *warfarin + moxifloxacin* pair. The most common pDDI was *warfarin + NSAIDs* (12,492 subjects; 7,581 with concomitant prescription and 2804 with co-prescriptions).

### Logistic regression analysis

Table **3** (and Table **S4**) shows the main results of the logistic regression analysis. With regard to **concomitant** events, male gender conferred an increased risk of pDDIs in 7 pairs for both cohorts of patients, and in 12 pairs for one of the two involved cohorts. The highest adjusted odds ratio (aOR) was present in the *omeprazole + clopidogrel* pair for patients in the *omeprazole* cohort (aOR 3.25, 95% CI 2.82-3.74). In 4 pairs -verapamil *+ atenolol*, *betablockers + verapamil*, *methotrexate +* (*NSAIDs or ASA*), and *SSRIs +* (*NSAIDs or ASA*)— male gender decreased the probability of pDDIs. The lowest value was observed in the *warfarin + levothyroxine* pair for the *warfarin* cohort (aOR 0.41, 95% CI 0.36-0.46). In most cases, the aOR values for both males and females were quite close to 1.

**Table 3:** Adjusted odds ratios (95% CI) of concomitant prescription or co-prescription for gender, age, and number of prescribed drugs.

	**Concomitant prescription**		**Co-prescription**	
	**aOR min**	**aOR max**	**aOR min**	**aOR max**
	**(95% CI)**	**(95% CI)**	**(95% CI)**	**(95% CI)**
**Female**	ref	ref	ref	ref
**Male**	0.41	3.25	0.38	3.46
	(0.36 - 0.46)	(2.82 - 3.74)	(0.32 -0.44)	(2.86 -4.17)
	for levothyroxine concomitance in warfarin cohort	for clopidogrel concomitance in omeprazole cohort	for levothyroxine coprescription in warfarin cohort	for clopidogrel coprescription in omeprazole cohort
**<50 years**	ref	ref	ref	ref
**50-64 years**	0.86	8.44	0.86	9.76
	(0.76 - 0.98)	(7.03 - 10.12)	(0.75 -0.99)	(7.18 -13.27)
	for metotrexate concomitance in NSAIDs-ASA cohort	for simvastatin concomitance in clarithromycin cohort	for metotrexate coprescription in NSAIDs-ASA cohort	for simvastatin coprescription in clarithromycin cohort
**65-74 years**	0.26	11.19	0.18	10.86
	(0.13 - 0.49)	(9.31 - 13.45)	(0.06 -0.51)	(7.94 -14.87)
	for itraconazole concomitance in warfarin cohort	for simvastatin concomitance in clarithromycin cohort	for itraconazole coprescription in warfarin cohort	for simvastatin coprescription in clarithromycin cohort
**≥75 years**	0.20	11.26	0.15	14.89
	(0.11 - 0.38)	(8.66 - 14.63)	(0.05 -0.42)	(10.43 -21.26)
	for itraconazole concomitance in warfarin cohort	for simvastatin concomitance in levothyroxine cohort	for itraconazole coprescription in warfarin cohort	for warfarin coprescription in levothyroxine cohort
**<5 drugs**	ref	ref	ref	ref
**5-9 drugs**	2.14	14.12	1.92	9.62
	(1.56 - 2.95)	(5.21 - 38.33)	(1.29 -2.87)	(3.51 -26.37)
	for SSRIs concomitance in warfarin cohort	for clopidogrel concomitance in omeprazole cohort	for SSRIs coprescription in warfarin cohort	for clopidogrel coprescription in omeprazole cohort
**≥10 drugs**	3.21	74.00	3.21	47.54
	(2.97 - 3.48)	(27.61 - 198.34)	(2.97 -3.48)	(17.67 -127.88)
	for enalapril concomitance in ASA cohort	for clopidogrel concomitance in omeprazole cohort	for ASA coprescription in enalapril cohort	for clopidogrel coprescription in omeprazole cohort

aOR: adjusted odds ratio

Generally, ages ≥50 years were associated with an increased risk; a clear trend was observed in 19 cohorts, with the strongest evidence in the *levothyroxine* cohort for *warfarin* concomitance (aORs 2.75 [95% CI 2.10-3.61], 6.99 [95% CI 5.38-9.07], and 11.26 [95% CI 8.66-14.63] in the three age groups, respectively, vs the <50 years class). On the other hand, in one cohort the aOR decreased with increasing age; this was the *NSAIDs or ASA* cohort for *methotrexate* concomitance (aORs 0.86 [95% CI 0.76-0.98], 0.74 [95% CI 0.56-0.73], and 0.33 [95% CI 0.28-0.39] in the three age groups, respectively). 

The number of drugs prescribed to the patient during the study period represented a very strong risk factor for pDDIs. *Omeprazole + clopidogrel* was the pair with most pronounced evidence (*omeprazole* cohort: aOR 14.12 [95% CI 5.21-38.33] for 5-9 drugs; aOR 74.00 [95% CI 27.61-198.34] for ≥10 drugs, vs <5 drugs; *clopidogrel* cohort: aOR 8.25 [95% CI 2.98-22.87]; aOR 21.65 [95% CI 7.91-59.28] for 5-9 and ≥10 drugs, respectively). Only in the *simvastatin + gemfibrozil* pair all aORs were not significant, also if >1.

For all analysed risk factors, the aORs for coprescription showed similar patterns, although with milder associations and not always reaching statistical significance (Table **3** and Table **S4**).

## Discussion

Only limited recent data are available on the prevalence of potential DDIs in Italy. The aim of this study was to estimate the prevalence of clinically relevant pDDIs in general practice among the approximately 2 million residents of the Basilicata and Marche regions, and to examine possible predictors of potential DDI exposure.

These analyses suggested that many patients have been exposed to the 27 drug combinations identified as clinically significant, well-documented and widely used in Italy; 8.5% of the study population received concomitant prescriptions and 6.0% received co-prescriptions of pDDIs. 

Considering persons with at least one potential DDI during the 20-month period, the prevalence estimates applied to the Italian population resulted in 5.02 million people with pDDIs (referred only to the 27 pairs), with females accounting for 2.89 million of these, and persons 65 years of age or older for 67.6%. The occurrence of pDDI events in Italy would be 69,259 million, with a ratio of 1 to 3 of co-prescription vs concomitant events.

Few other studies have evaluated pDDIs in general practice. A retrospective follow-up study of outpatient prescription data in Italy found that among more than 4 million subjects, 8,894 clinically important potential DDIs were identified, representing a 1-year period prevalence of 211 per 100,000 individuals [21]. A register analysis study in general practice carried out in Denmark found that 6% of the population were exposed to potential drug interactions during a 1-year period [22]. In a retrospective analysis of the clinical records from 16 general practitioners in an Italian region, 119 unique severe potential DDIs occurred 1,037 times in 758 patients (4.7% of the total number of patients) [23]. Previous studies showed that 0.5–4.0% of patients are exposed to serious potential drug interactions in primary health care [1,24,25]. Differences in the selection of DDIs could explain the differences in the estimated rates. Many lists of potentially interacting drugs are available [26–29], and while there is general agreement on their documentation and clinical relevance, their clinical and economic burden at the population level strongly depends on the drugs in the market at a national level and on prescribing patterns in each local context; this means that comparisons among different settings are most likely irrelevant.

Nevertheless, the evidence from this and other similar studies, as well as those derived from hospitals [30,31] and emergency departments [32,33], show that the pDDIs are a major issue. 

In recent years, an enormous quantity of data on drug interactions has been published. Although it is likely that pDDIs are common, only a few of these induce serious adverse events and often only in predisposed patients [5,33]. Indeed, in considering the incidence of DDIs, we should distinguish between potential interactions and interactions that actually result in clinically adverse effects. In one study [34] of 2,422 patients studied over a period of two months, 113 (4.7%) were taking drugs that could potentially interact, but only seven cases showed any clinical evidence of interaction (0.3% of all the patients; 6.2% of those potentially affected). In a French study [35] overlooking contraindication to the concomitant drug use was the most frequent feature in the cases of non-respect of the Summary of Product Characteristics (38%), but it was rarely the cause of an adverse drug reaction (6%). Other studies have also shown that fewer than 11% of the potential interactions identified for a prescription resulted in an adverse reaction, and these were rarely the reason for hospital admission [36,37]. In a review on hospitalisations and emergency department visits due to drug–drug interactions [38], DDIs were responsible for 0.05% of the Emergency Department visits, 0.57% of the admissions and 0.12% of the re-hospitalisations. We should note that these percentages may be an underestimation, because it is possible that medical practitioners and pharmacists did not recognise adverse patient outcomes caused by DDIs [39,40]. 

Although the percentages are modest, the number of adverse outcomes due to DDIs is substantial, because of the large numbers of ED visits and (re-)hospitalisations. Moreover, with certain combinations of drugs, there can be consequences that are very rare but are clinically relevant, or less harmful consequences that arise more frequently. In both cases, the overall burden increases with the number of drug users. For example, in our analysis, NSAIDs—a widely used therapeutic class [41] involved in seven of the selected pairs—showed the highest case-exposure rate in combination with ACE inhibitors (6,253.4/100,000 PP). NSAIDs interact with different groups of antihypertensive drugs [42,43], reducing their antihypertensive activity. Although the changes in blood pressure resulting from this interaction are typically small, some patients can experience substantial elevations in both systolic and diastolic blood pressure. A USA study estimated that avoiding minor changes in systolic pressure in patients with osteoarthritis on treatment with NSAIDs would have prevented over 30,000 deaths due to stroke, and over 2000 deaths due to coronary disease [44].

Antibiotics are widely used in Italy, especially those belonging to the class of fluoroquinolones [41]. Blood glucose alterations may occur with fluoroquinolones at a higher incidence than was initially believed [45]. This could be a significant problem for high-risk patients such as diabetics. In our analysis, patients exposed to concomitant prescriptions of metformin and a fluoroquinolone were 428.6/100,000 PP, implying the need for close monitoring of blood glucose in these subjects.

Seven of the selected potential DDI types involved warfarin. Given the narrow International Normalised Ratio (INR) range in which patients should be maintained, even slight increases or decreases in drug concentration in the plasma could have clinically relevant effects. On the other hand, for the same reasons, patients treated with warfarin are strictly monitored, and drug doses are adjusted in accordance with changes in the INR. Since we have no information about the actual administered doses, it is possible that the high prevalence of potential DDIs involving warfarin in our observations has not a high clinical burden. 

The only selected pair of drugs with contraindicated concomitant use, *simvastatin + itraconazole*, showed a very low prevalence in the population (15.8/100,000 PP). The use of itraconazole is not common in primary care, and it is usually administered under specialist care, meaning that few patients are exposed to potentially adverse events resulting from these drug combinations.

Whether two interacting drugs can be used at the same time without serious consequences depends on whether the benefit of both drug therapies outweighs the risk of the DDI, taking into account the availability of alternatives. Our survey assessed separately concomitant prescription and co-prescription. This second case—i.e., the prescription of two potentially interacting drugs on the same day—addresses actual prescriber intention. The intentional act of co-prescribing may reflect a conscious choice, driven by the absence of therapeutic alternatives or accompanied by clear instructions and recommendations to the patient, but it may also indicate a lack of knowledge and preparation on the part of the physician. A survey of prescribers with the Southern California Veterans Affairs Healthcare System found that clinicians correctly identified only 44% of DDIs [9]. Studies aimed at evaluating pharmacists’ knowledge have also found a low recall of DDIs [46,47]. One factor that may complicate health professionals’ ability to detect DDIs is that the number of possible interactions increases as the number of medications a patient is taking increases [48]; prescribers should recognise that patients often come to them medicated with several drugs, often acquired from multiple sources (e.g., over-the-counter and from other prescribers). Anyhow, physicians cannot be expected to know all of the huge number of pharmaceuticals available and their potential for drug interactions. Computerised drug prescribing alerts could help them, but are often overridden because of poor specificity and overload [49,50]. If pharmacists are careful to check the dispensed drugs and have a good relationship with the prescriber, they may help to counteract this issue, protecting patient safety and increasing physician's awareness.

In our analyses, the case-exposure rates of co-prescriptions were greatest for *ACE inhibitors + NSAIDs or ASA* (4,620.6/100,000 PP), *SSRIs + NSAIDs or ASA* (884.6/100,000 PP), and enalapril + *ASA* (517.4/100,000 PP). These data illustrate the extent of the problem of interaction faced by physicians in managing chronic therapies.

We also assessed the extent of the association between some factors and the risk of having a pDDI in the pairs included in the study. In the regression analyses performed for the two cohorts of exposed patients for each drug pairs (overall 54 analyses), the male gender was a risk factor in 25 analyses (with a maximum aOR of 3.25 in the *omeprazole* cohort for concomitant *clopidogrel*), an age of ≥75 years was a risk factor in 36 analyses (with a maximum aOR of 11.26 in the *levothyroxine* cohort for concomitant warfarin), and having 10 or more other prescribed drugs was a risk factor in 50 analyses (with a maximum aOR of 74.00 in the *omeprazole* cohort for concomitant *clopidogrel*). Consistent with these results, Cruciol-Souza et al. [51] found that the odds of exposure to potential DDIs were significantly higher in patients aged ≥55 years (OR 1.41) and in those who had been administered more than 7 drugs (OR 9.91). They found, however, that odds of exposure were higher among females (OR 1.23). This difference could depend on list of pDDIs choice, or from differences in prescribing habits. In the study by Bjerrum et al., patient-related factors associated with the increased risk of potential drug interactions were a high age and a high number of concurrently used drugs [22]. Gagne et al. found that the odds of exposure were highest among those aged 65 years or older, males, and those with more chronic conditions. The odds of exposure increased by 1.39 times with each addition of a prescription medication [21]. As expected, older age and polytherapy, two conditions that are closely related, greatly increase the risk of interaction; these factors may be used by physicians to identify fragile patients who should receive maximum attention to the potential for DDIs. 

The major limitation of our study is that we did not assess the harm associated with potentially hazardous interactions, and therefore the exposure rates may overestimate the real clinical impact of DDIs. It was also not possible to determine whether the choice of drugs was deliberate, and whether it was preceded by a careful evaluation of the risk-benefit ratio (including available treatment options and therapeutic goals) and was accompanied by proper instructions to the patient, to minimise the risk of adverse events. Furthermore, our study was limited by the number and type of interactions evaluated, and by the dataset. First, different results may have been obtained, to the extent that other interactions were selected. Second, similar to many other databases, ASSET depends on the Italian reimbursement system. Consequently, we may not have recognised other potentially serious interactions, and we could have underestimated the actual magnitude of some selected pDDIs (e.g., those involving NSAIDs), since only NHS-covered prescribed medications were included and not OTC non-prescription medications, or herbal and/or home remedies. Drugs dispensed in hospitals and nursing homes were also not included in the study. In addition, the estimated time of prescription coverage based on the DDDs actually provides only a rough evaluation of the timing of use. Finally, we do not know whether the prescribed medicines were taken. This limitation, however, is less relevant if we look at the results from the point of view of the appropriateness of the prescription habits of physicians.

## Conclusions

Limited to the 27 selected drugs pairs, we observed that males, elderly and people taking multiple medications were more frequently exposed to a concomitant or co-prescription of potentially interacting drugs. These results can guide the interventions by health authorities, aimed at containing the epidemiological impact of the pDDIs. On the other hand, the different rates of exposure in the two genders or in the age classes do not involve less attention by the physician in respect of those subjects that showed a lower probability of being exposed to pDDIs. Most importantly, clinicians should recognise that each medication added to a therapeutic regimen significantly increase the risk of patients' exposure to potential DDIs.

The noticeably high number of subjects exposed to pDDIs —with many of them experiencing multiple episodes during the observation period, and many taking more than one pair of interacting drugs— should encourage health authorities to develop new effective strategies, since the computerised tools available to support the prescription process are not generally used by physicians. We suggest, for example, that an institutional Committee of experts outline a list of DDIs based both on the clinical relevance of the interaction and on the prevalence of drug use, thus defining an indicator of appropriate prescribing; this should be evaluated by all regions to have a complete national picture on which educational prescriber-targeted strategies can be drawn.

## Supporting Information

Table S1 **Clinical relevance, documentation and level of use of chosen potential Drug–Drug Interactions.**
(DOCX)

Table S2 **Characteristics of cohorts exposed to potential Drug–Drug Interactions.**
(DOCX)

Table S3 **Event rates in patients with concomitant prescriptions and in patients with co-prescriptions.**
(DOCX)

Table S4 **Logistic regression (panel A: Concomitant prescriptions; panel B: Co-prescriptions).**
(DOCX)
