# Correction: Prevalence of the Prescription of Potentially Interacting Drugs

**DOI:** 10.1371/annotation/01652378-8216-4387-9b89-a43429707cae

**Published:** 2013-11-08

**Authors:** Elena Tragni, Manuela Casula, Vasco Pieri, Giampiero Favato, Alberico Marcobelli, Maria Giovanna Trotta, Alberico Luigi Catapano

Affiliation number 1 for the first, second, third, fourth and sixth authors is incorrect. The correct affiliation is: Epidemiology and Preventive Pharmacology Centre (SEFAP), Department of Pharmacological and Biomolecular Sciences (DiSFeB), University of Milan, Italy.

