# Supplementary material for: Prevalence of the Prescription of Potentially Interacting Drugs
Source: PLoS One. 2013 Oct 11;8(10):e78827. doi: 10.1371/journal.pone.0078827 (PMC3795676; doi:10.1371/journal.pone.0078827)
Supplement: Table S1 — Clinical relevance, documentation and level of use of chosen potential Drug–Drug Interactions. (DOCX) [file pone.0078827.s001.docx]

**Table S1.** Clinical relevance, documentation and level of use of chosen potential Drug–Drug Interactions.

| **pDDI** | | **CLINICAL RELEVANCE (*)** | **DOCUMENTATION (*)** | **EFFECTS (*)** | **RANK USE (●)** |
| --- | --- | --- | --- | --- | --- |
| **1** | **Simvastatin** | Contraindicated | Good | increased risk of myopathy or rhabdomyolisys | 7 |
|  | **Itraconazole** |  |  |  | 103 |
| **2** | **Metformin** | Major | Excellent | changes in blood glucose and increased risk  of hypoglycaemia or hyperglycaemia | 22 |
|  | **Fluoroquinolones** (10 molecules) |  |  |  | class |
| **3** | **Omeprazole** | Major | Excellent | increased risk for thrombosis | 13 |
|  | **Clopidogrel** |  |  |  | 102 |
| **4** | **Warfarin** | Major | Excellent | increased risk of bleeding | 50 |
|  | **Amiodarone** |  |  |  | 64 |
| **5** | **Warfarin** | Major | Excellent | increased risk of bleeding | 50 |
|  | **Moxifloxacin** |  |  |  | >100 |
| **6** | **Simvastatin** | Major | Excellent | increased risk of myopathy or rhabdomyolisys | 7 |
|  | **Amiodarone** |  |  |  | 64 |
| **7** | **Warfarin** | Major | Excellent | increased risk of bleeding and rhabdomyolisys | 50 |
|  | **Simvastatin** |  |  |  | 7 |
| **8** | **Digoxin** | Major | Excellent | increased serum digoxin concentrations and toxicity  (nausea, vomiting, arrhythmias). | 44 |
|  | **Verapamil** |  |  |  | 63 |
| **9** | **Warfarin** | Major | Good | increased risk of bleeding | 50 |
|  | **SSRIs** (6 molecules) |  |  |  | class |
| **10** | **Verapamil** | Major | Good | hypotension, bradycardia | 63 |
|  | **Atenolol** |  |  |  | 11 |
| **11** | **Eparines** (10 molecules) | Major | Good | increased risk of bleeding | class |
|  | **Nimesulide, indomethacin, or acetylsalicylic acid** |  |  |  | 17; >100; 1 |
| **12** | **Amiodarone** | Major | Good | increased risk of cardiotoxicity  (QT prolongation, torsades de pointes, cardiac arrest) | 64 |
|  | **Antiarythmics Ia** (3 molecules) |  |  |  | class |
| **13** | **Methotrexate** | Major | Good | increased risk of methotrexate toxicity | >100 |
|  | **Omeprazole** |  |  |  | 13 |
| **14** | **Simvastatin** | Major | Good | increased risk of myopathy or rhabdomyolisys | 7 |
|  | **Gemfibrozil** |  |  |  | 67 |
| **15** | **Simvastatin** | Major | Good | increased risk of myopathy or rhabdomyolisys | 7 |
|  | **Clarithromycin** |  |  |  | 71 |
| **16** | **Betablockers** (14 molecules) | Major | Good | hypotension, bradycardia | class |
|  | **Verapamil** |  |  |  | 63 |
| **17** | **Simvastatin** | Major | Good | increased risk of myopathy or rhabdomyolisys | 7 |
|  | **Verapamil** |  |  |  | 63 |
| **18** | **Enalapril** | Major | Fair | hypersensitivity reactions (Stevens-Johnson syndrome,  skin eruptions, anaphylactic coronary spasm) | 5 |
|  | **Allopurinol** |  |  |  | >100 |
| **19** | **Warfarin** | Major/moderate | Excellent/good/fair | increased risk of bleeding | 50 |
|  | **NSAIDs or ASA** (26 molecules) |  |  |  | class |
| **20** | **Methotrexate** | Major/moderate | Good/fair | increased risk of methotrexate toxicity (leukopenia, thrombocytopenia, anaemia, nephrotoxicity, mucosal ulcerations) | >100 |
|  | **NSAIDs or ASA** (26 molecules) |  |  |  | class |
| **21** | **Enalapril** | Moderate | Excellent | decreased effectiveness of enalapril | 5 |
|  | **ASA** |  |  |  | 1 |
| **22** | **Enalapril** | Moderate | Good | hyperkalemic lactic acidosis | 5 |
|  | **Metformin** |  |  |  | 22 |
| **23** | **Warfarin** | Moderate | Good | increased risk of bleeding | 50 |
|  | **Itraconazole** |  |  |  | 103 |
| **24** | **Warfarin** | Moderate | Good | increased risk of bleeding | 50 |
|  | **Levotiroxine** |  |  |  | 9 |
| **25** | **Simvastatin** | Moderate | Good | increased risk of digoxin toxicity (nausea, vomiting, arrhythmias) | 7 |
|  | **Digoxin** |  |  |  | 44 |
| **26** | **ACE inhibitors** (25 molecules) | Moderate | Good | decreased antihypertensive and natriuretic effects | class |
|  | **NSAIDs or ASA** (26 molecules) |  |  |  | class |
| **27** | **SSRIs** (6 molecules) | Moderate | Good | increased risk of bleeding | class |
|  | **NSAIDs or ASA** (26 molecules) |  |  |  | class |

(*) Micromedex, 2009

(●) Osmed, 2004 (DDD/1000 inhabitants daily)
