# Supplementary material for: Prevalence of the Prescription of Potentially Interacting Drugs
Source: PLoS One. 2013 Oct 11;8(10):e78827. doi: 10.1371/journal.pone.0078827 (PMC3795676; doi:10.1371/journal.pone.0078827)
Supplement: Table S2 — Characteristics of cohorts exposed to potential Drug–Drug Interactions. (DOCX) [file pone.0078827.s002.docx]

**Table S2.** Characteristics of cohorts exposed to potential Drug–Drug Interactions.

| **pDDI** | | | **Patients** | **CoP/ConcoP** | **Male** | **Age (years)** | **N drugs** | **Events/subjects** |
| --- | --- | --- | --- | --- | --- | --- | --- | --- |
|  |  |  | N |  | N (%) | mean±SD | mean±SD | mean±SD |
| **1** | **Simvastatin - Itraconazole** | **ConcoP** | 326 |  | 156 (47.9) | 63.5±12.1 | 14.3±8.3 | 2.1±2.6 |
|  | (N 44,071) - (N 39,872) | **CoP** | 149 | 0,46 | 77 (51.7) | 63.4±11.1 | 14.1±6.7 | 1.4±1.3 |
| **2** | **Metformin - Fluoroquinolones** | **ConcoP** | 8,845 |  | 4,152 (46.9) | 67.7±11.4 | 14.0±6.8 | 2.2±2.2 |
|  | (N 57,575) - (N 268,825) | **CoP** | 3,898 | 0,44 | 1,801 (46.2) | 68.0±11.5 | 14.1±7.0 | 1.5±1.2 |
| **3** | **Omeprazol-Clopidogrel** | **ConcoP** | 1,020 |  | 755 (74.0) | 64.5±11.8 | 16.4±7.1 | 9.0±8.6 |
|  | (N 66,207) - (N 3,330) | **CoP** | 597 | 0,59 | 450 (75.4) | 63.4±12.2 | 15.7±7.2 | 2.7±2.1 |
| **4** | **Warfarin - Amiodarone** | **ConcoP** | 3,126 |  | 1,876 (60.0) | 70.6±10.7 | 14.0±6.4 | 5.9±5.4 |
|  | (N 22,251) - (N 13,327) | **CoP** | 2,502 | 0,80 | 1,484 (59.3) | 70.3±10.7 | 13.7±6.4 | 2.9±2.5 |
| **5** | **Warfarin - Moxifloxacin** | **ConcoP** | 579 |  | 352 (60.8) | 71.7±10.3 | 16.7±9.0 | 1.5±0.5 |
|  | (N 22,251) - (N 51,224) | **CoP** | 141 | 0,24 | 83 (58.7) | 71.7±11.3 | 17.0±13.6 | 1.1±0.5 |
| **6** | **Simvastatin - Amiodarone** | **ConcoP** | 1,384 |  | 864 (62.4) | 71.2±10.7 | 15.7±7.2 | 10.5±10.5 |
|  | (N 44,071) - (N 13,327) | **CoP** | 1,094 | 0,79 | 687 (62.8) | 71.3±10.6 | 15.5±7.1 | 4.3±3.7 |
| **7** | **Warfarin - Simvastatin** | **ConcoP** | 1,841 |  | 1,055 (57.3) | 70.5±9.9 | 14.3±6.7 | 8.9±8.9 |
|  | (N 22,251) - (N 44,071) | **CoP** | 1,462 | 0,79 | 846 (57.9) | 70.3±9.9 | 14.0±6.6 | 3.9±3.4 |
| **8** | **Digoxin - Verapamil** | **ConcoP** | 1,579 |  | 556 (35.2) | 75.4±11.6 | 13.9±6.9 | 9.5±9.6 |
|  | (N 39,466) - (N 13,654) | **CoP** | 1,276 | 0,81 | 438 (34.3) | 75.3±11.8 | 13.3±6.5 | 5.7±4.9 |
| **9** | **Warfarin - SSRIs** | **ConcoP** | 1,635 |  | 697 (42.6) | 71.1±11.9 | 14.9±7.8 | 5.8±7.3 |
|  | (N 22,251) - (N 95,811) | **CoP** | 938 | 0,57 | 380 (40.5) | 71.4±12.0 | 14.2±6.7 | 2.9±2.6 |
| **10** | **Verapamil - Atenolol** | **ConcoP** | 300 |  | 100 (33.3) | 64.8±12.5 | 13.8±8.2 | 3.1±4.7 |
|  | (N 13,654) - (N 40,812) | **CoP** | 83 | 0,28 | 28 (33.7) | 64.8±13.4 | 13.1±6.2 | 2.1±2.2 |
| **11** | **Eparines - (Nimesulide, Indomethacin, or Acetylsalicylic acid)** | **ConcoP** | 18,778 |  | 7,116 (37.9) | 71.9±12.9 | 13.8±7.0 | 2.8±4.0 |
|  | (N 81,895) - (N 309,296) | **CoP** | 7,214 | 0,38 | 2,563 (35.5) | 71.0±14.0 | 13.4±7.2 | 1.4±1.2 |
| **12** | **Amiodarone - Antiarythmics Ia** | **ConcoP** | 55 |  | 28 (50.9) | 70.6±13.3 | 17.3±16.4 | 2.8±4.7 |
|  | (N 15,786) - (N 2,617) | **CoP** | 16 | 0,29 | 10 (62.5) | 65.7±15.9 | 11.8±5.5 | 3.4±4.4 |
| **13** | **Methotrexate - Omeprazole** | **ConcoP** | 423 |  | 141 (33.3) | 59.9±15.4 | 14.7±6.8 | 9.5±10.9 |
|  | (N 2,786) - (N 66,207) | **CoP** | 291 | 0,69 | 98 (33.7) | 59.8±15.1 | 14.3±6.5 | 3.0±2.8 |
| **14** | **Simvastatin - Gemfibrozil** | **ConcoP** | 61 |  | 38 (62.3) | 65.5±10.7 | 13.4±7.3 | 3.3±5.0 |
|  | (N 44,071) - (N 2,314) | **CoP** | 23 | 0,38 | 17 (73.91) | 64.5±13.0 | 11.9±6.2 | 2.6±2.5 |
| **15** | **Simvastatin - Clarithromycin** | **ConcoP** | 2,020 |  | 991 (49.1) | 66.2±11.6 | 13.9±6.5 | 1.6±1.2 |
|  | (N 44,071) - (N 185,243) | **CoP** | 700 | 0,35 | 322 (46.0) | 65.5±11.1 | 13.6±6.2 | 1.2±0.7 |
| **16** | **Betablockers - Verapamil** | **ConcoP** | 1,128 |  | 414 (36.7) | 67.1±12.2 | 14.0±7.5 | 3.7±6.4 |
|  | (N 127,947) - (N 13,654) | **CoP** | 354 | 0,31 | 127 (35.9) | 66.8±13.3 | 13.3±6.6 | 2.8±3.3 |
| **17** | **Simvastatin - Verapamil** | **ConcoP** | 922 |  | 392 (42.5) | 70.5±11.0 | 13.9±6.9 | 12.0±13.4 |
|  | (N 44,071) - (N 13,654) | **CoP** | 726 | 0,79 | 313 (43.1) | 70.5±11.2 | 13.3±6.6 | 5.7±5.2 |
| **18** | **Enalapril - Allopurinol** | **ConcoP** | 4,206 |  | 2,611 (62.1) | 72.8±11.3 | 13.5±6.9 | 8.2±9.6 |
|  | (N 45,279) - (N 52,738) | **CoP** | 2,841 | 0,68 | 1,794 (63.1) | 72.7±11.4 | 13.1±6.6 | 3.3±3.1 |
| **19** | **Warfarin - (NSAIDs or ASA)** | **ConcoP** | 7,581 |  | 3,898 (51.4) | 71.5±10.6 | 14.8±6.7 | 3.0±4.0 |
|  | (N 22,251) - (N 549,362) | **CoP** | 2,804 | 0,37 | 1,370 (48.9) | 71.4±10.8 | 14.4±6.8 | 1.9±1.9 |
| **20** | **Methotrexate - (NSAIDs or ASA)** | **ConcoP** | 1,797 |  | 566 (31.5) | 59.1±15.6 | 12.7±6.0 | 7.7±8.3 |
|  | (N 2,786) - (N 549,362) | **CoP** | 1,395 | 0,78 | 430 (30.8) | 58.6±15.8 | 12.5±6.2 | 3.6±3.2 |
| **21** | **Enalapril - ASA** | **ConcoP** | 13,660 |  | 6,974 (51.1) | 71.9±12.1 | 12.3±6.4 | 15.4±16.7 |
|  | (N 45,279) - (N 171,613) | **CoP** | 10,676 | 0,78 | 5,511 (51.6) | 71.8±12.2 | 11.9±6.2 | 5.0±4.3 |
| **22** | **Enalapril - Metformin** | **ConcoP** | 3,835 |  | 1,818 (47.4) | 68.7±11.4 | 12.9±6.9 | 16.8±19.7 |
|  | (N 45,279) - (N 57,575) | **CoP** | 2,988 | 0,78 | 1,399 (46.8) | 68.6±11.4 | 12.5±6.4 | 6.0±5.5 |
| **23** | **Warfarin - Itraconazole** | **ConcoP** | 96 |  | 44 (45.8) | 64.4±14.5 | 15.1±7.3 | 1.5±1.2 |
|  | (N 22,251) - (N 39,872) | **CoP** | 32 | 0,33 | 17 (53.1) | 63.7±13.4 | 13.8±6.4 | 1.1±0.3 |
| **24** | **Warfarin - Levothiroxine** | **ConcoP** | 1,227 |  | 395 (32.2) | 69.7±11.3 | 14.6±7.3 | 8.1±8.0 |
|  | (N 22,251) - (N 65,478) | **CoP** | 788 | 0,64 | 238 (30.2) | 70.0±11.0 | 13.9±6.4 | 3.0±2.3 |
| **25** | **Simvastatin - Digoxin** | **ConcoP** | 1,953 |  | 942 (48.2) | 73.6±10.1 | 15.6±7.1 | 8.3±9.0 |
|  | (N 44,071) - (N 39,466) | **CoP** | 1,420 | 0,73 | 685 (48.2) | 73.8±9.8 | 15.2±6.8 | 4.5±3.9 |
| **26** | **ACE inhibitors - (NSAIDs or ASA)** | **ConcoP** | 129,037 |  | 57,852 (44.8) | 69.5±12.4 | 11.2±6.0 | 13.3±16.9 |
|  | (N 239,098) - (N 549,362) | **CoP** | 95,344 | 0,74 | 42,912 (45.0) | 70.0±12.3 | 11.3±6.0 | 4.6±4.5 |
| **27** | **SSRIs - (NSAIDs or ASA)** | **ConcoP** | 32,803 |  | 10,186 (31.1) | 65.0±15.0 | 12.3±6.6 | 6.1±9.1 |
|  | (N 95,811) - (N 549,362) | **CoP** | 18,254 | 0,56 | 5,494 (30.1) | 66.1±14.6 | 12.4±6.7 | 2.8±3.0 |

ConcoP: patients with concomitant prescriptions

CoP: patients with coprescriptions
