# Supplementary material for: Prevalence of the Prescription of Potentially Interacting Drugs
Source: PLoS One. 2013 Oct 11;8(10):e78827. doi: 10.1371/journal.pone.0078827 (PMC3795676; doi:10.1371/journal.pone.0078827)
Supplement: Table S3 — Event rates in patients with concomitant prescriptions and in patients with co-prescriptions. (DOCX) [file pone.0078827.s003.docx]

**Table S3.** Event rates in patients with concomitant prescriptions and in patients with co-prescriptions.

| **pDDIs (drug A-drug B)** | | **Cases per 1,000 exposed* plan participants** | | **Cases per 100,000 plan participants** | |
| --- | --- | --- | --- | --- | --- |
|  |  | **with concomitant prescriptions** | **with coprescriptions** | **with concomitant prescriptions** | **with coprescriptions** |
| **1** | **Simvastatin-Itraconazole** | 7; 8 | 3; 4 | 15.8 | 7.2 |
| **2** | **Metformin-Fluoroquinolones** | 154; 33 | 68; 15 | 428.6 | 188.9 |
| **3** | **Omeprazol-Clopidogrel** | 15; 306 | 9; 179 | 49.4 | 28.9 |
| **4** | **Warfarin-Amiodarone** | 140; 235 | 112; 188 | 151.5 | 121.3 |
| **5** | **Warfarin-Moxifloxacin** | 26; 11 | 6; 3 | 28.1 | 6.8 |
| **6** | **Simvastatin-Amiodarone** | 31;104 | 25; 82 | 67.1 | 53.0 |
| **7** | **Warfarin-Simvastatin** | 83; 42 | 66; 33 | 89.2 | 70.9 |
| **8** | **Digoxin-Verapamil** | 40; 116 | 32; 93 | 76.5 | 61.8 |
| **9** | **Warfarin-ssris** | 73; 17 | 42; 10 | 79.2 | 45.5 |
| **10** | **Verapamil-Atenolol** | 22; 7 | 6; 2 | 14.5 | 4.0 |
| **11** | **Eparines-(Nimesulide, Indomethacin, or Acetylsalicylic acid)** | 229; 61 | 88; 23 | 910.0 | 349.6 |
| **12** | **Amiodarone-Antiarythmics Ia** | 3; 21 | 1; 6 | 2.7 | 0.8 |
| **13** | **Methotrexate-Omeprazole** | 152; 6 | 104; 4 | 20.5 | 14.1 |
| **14** | **Simvastatin-Gemfibrozil** | 1; 26 | 1; 10 | 3.0 | 1.1 |
| **15** | **Simvastatin-Clarithromycin** | 46; 11 | 16; 4 | 97.9 | 33.9 |
| **16** | **Betablockers-Verapamil** | 9; 83 | 3; 26 | 54.7 | 17.2 |
| **17** | **Simvastatin-Verapamil** | 21; 68 | 16; 53 | 44.7 | 35.2 |
| **18** | **Enalapril-Allopurinol** | 93; 80 | 63; 54 | 203.8 | 137.7 |
| **19** | **Warfarin-(nsaids or asa)** | 340; 14 | 126; 5 | 367.4 | 135.9 |
| **20** | **Methotrexate-(nsaids or asa)** | 645; 3 | 501; 3 | 87.1 | 67.6 |
| **21** | **Enalapril-asa** | 302; 80 | 236; 62 | 662.0 | 517.4 |
| **22** | **Enalapril-Metformin** | 85; 67 | 66; 52 | 185.9 | 144.8 |
| **23** | **Warfarin-Itraconazole** | 26; 2 | 6; 1 | 4.7 | 1.6 |
| **24** | **Warfarin-Levotiroxine** | 55; 19 | 35;12 | 59.5 | 38.2 |
| **25** | **Simvastatin-Digoxin** | 44;49 | 32;36 | 94.7 | 68.8 |
| **26** | **ace inhibitors-(nsaids or asa)** | 540; 235 | 399;174 | 6,253.4 | 4,620.6 |
| **27** | **Ssris-(nsaids or asa)** | 342; 60 | 191; 33 | 1,589.7 | 884.6 |

***** plan participants exposed to drug A (first number) and to drug B (second number)

**Concomitant Cases** are plan participants who received both drugs without regard to order or dosage whose coverage periods are partially operlapping

**Coprescription Cases** are plan participants who received both drugs in the same day.
