# Supplementary material for: Prevalence of the Prescription of Potentially Interacting Drugs
Source: PLoS One. 2013 Oct 11;8(10):e78827. doi: 10.1371/journal.pone.0078827 (PMC3795676; doi:10.1371/journal.pone.0078827)
Supplement: Table S4 — Logistic regression (panel A: Concomitant prescriptions; panel B: Co-prescriptions). (DOCX) [file pone.0078827.s004.docx]

**Table S4.** Logistic regression (panel A: Concomitant prescriptions; panel B: Co-prescriptions).

| **PANEL A** | | **Adjusted ORs (95% CI)** | | | | | |
| --- | --- | --- | --- | --- | --- | --- | --- |
|  |  | **SEX** | **AGE (years)** | | | **NUMBER of drugs** | |
|  |  | **male** | **50-64** | **65-74** | **≥75** | **5-9** | **≥10** |
| **1** | **Simvastatin** | 0.888 (0.713-1.106) | 0.891 (0.619-1.282) | 0.677 (0.468-0.979) | 0.443 (0.294-0.669) | 2.324 (1.388-3.891) | 4.648 (2.815-7.676) |
|  | **Itraconazole** | 1.555 (1.246-1.942) | 5.808 (4.027-8.376) | 10.120 (6.932-14.774) | 7.152 (4.688-10.912) | 4.223 (2.522-7.072) | 9.441 (5.675-15.704) |
| **2** | **Metformin** | 1.066 (1.017-1.118) | 1.602 (1.453-1.766) | 1.731 (1.571-1.906) | 1.570 (1.422-1.734) | 3.529 (3.116-3.998) | 9.483 (8.400-10.706) |
|  | **Fluoroquinolones** | 1.069 (1.024-1.116) | 4.337 (3.954-4.756) | 5.050 (4.607-5.537) | 3.579 (3.257-3.933) | 3.959 (3.502-4.476) | 10.189 (9.040-11.484) |
| **3** | **Omeprazole** | 3.246 (2.817-3.740) | 1.817 (1.464-2.255) | 1.281 (1.027-1.598) | 0.878 (0.696-1.106) | 14.124 (5.205-38.326) | 74.001 (27.610-198.34) |
|  | **Clopidogrel** | 1.267 (1.066-1.505) | 0.988 (0.757-1.290) | 0.791 (0.602-1.038) | 0.643 (0.484-0.856) | 8.253 (2.979-22.865) | 21.650 (7.907-59.282) |
| **4** | **Warfarin** | 1.481 (1.370-1.602) | 1.942 (1.590-2.370) | 1.851 (1.530-2.239) | 1.540 (1.274-1.862) | 3.824 (2.904-5.035) | 7.015 (5.353-9.194) |
|  | **Amiodarone** | 1.153 (1.061-1.253) | 2.269 (1.837-2.802) | 1.996 (1.633-2.439) | 1.217 (0.998-1.485) | 4.008 (3.021-5.318) | 5.428 (4.109-7.170) |
| **5** | **Warfarin** | 1.486 (1.254-1.762) | 1.832 (1.109-3.027) | 1.727 (1.066-2.797) | 1.750 (1.085-2.822) | 4.347 (1.749-10.804) | 14.576 (5.974-35.568) |
|  | **Moxifloxacin** | 1.530 (1.292-1.811) | 4.193 (2.548-6.900) | 7.028 (4.344-11.369) | 8.632 (5.353-13.918) | 7.006 (2.826-17.370) | 24.840 (10.188-60.566) |
| **6** | **Simvastatin** | 1.910 (1.707-2.137) | 1.449 (1.063-1.977) | 2.146 (1.592-2.892) | 3.404 (2.528-4.584) | 4.582 (2.748-7.642) | 15.810 (9.587-26.070) |
|  | **Amiodarone** | 1.267 (1.127-1.423) | 1.982 (1.434-2.738) | 1.736 (1.273-2.367) | 1.252 (0.920-1.703) | 3.817 (2.271-6.414) | 8.470 (5.091-14.092) |
| **7** | **Warfarin** | 1.261 (1.144-1.391) | 2.440 (1.857-3.207) | 2.289 (1.758-2.979) | 1.688 (1.296-2.198) | 2.928 (2.075-4.133) | 5.983 (4.274-8.377) |
|  | **Simvastatin** | 1.511 (1.373-1.663) | 1.769 (1.352-2.313) | 2.662 (2.053-3.451) | 3.449 (2.657-4.478) | 3.460 (2.459-4.867) | 8.457 (6.061-11.800) |
| **8** | **Digoxin** | 0.748 (0.673-0.832) | 2.199 (1.579-3.063) | 2.486 (1.827-3.382) | 1.979 (1.469-2.665) | 3.001 (2.100-4.289) | 4.102 (2.887-5.828) |
|  | **Verapamil** | 0.915 (0.817-1.025) | 1.447 (1.037-2.019) | 2.178 (1.600-2.965) | 4.379 (3.248-5.903) | 4.638 (3.245-6.628) | 8.828 (6.225-12.519) |
| **9** | **Warfarin** | 0.677 (0.611-0.750) | 1.055 (0.821-1.356) | 1.220 (0.968-1.536) | 1.188 (0.947-1.492) | 2.144 (1.555-2.954) | 4.469 (3.272-6.102) |
|  | **SSRIs** | 1.569 (1.419-1.734) | 2.472 (1.941-3.148) | 6.434 (5.147-8.042) | 7.857 (6.304-9.793) | 3.512 (2.562-4.815) | 9.050 (6.662-12.292) |
| **10** | **Verapamil** | 0.734 (0.575-0.938) | 1.078 (0.720-1.613) | 0.719 (0.484-1.067) | 0.322 (0.211-0.490) | 5.718 (2.745-11.912) | 10.808 (5.249-22.251) |
|  | **Atenolol** | 0.772 (0.606-0.983) | 0.898 (0.607-1.329) | 1.156 (0.788-1.697) | 1.113 (0.736-1.682) | 5.560 (2.683-11.52) | 14.542 (7.126-29.679) |
| **11** | **Eparines** | 1.201 (1.159-1.245) | 2.216 (2.057-2.387) | 3.430 (3.194-3.684) | 4.074 (3.801-4.367) | 3.633 (3.333-3.960) | 7.346 (6.749-7.994) |
|  | **Nimesulide, Indomethacin, or Acetylsalicylic acid** | 0.782 (0.759-0.807) | 1.483 (1.383-1.590) | 2.284 (2.139-2.439) | 3.353 (3.145-3.574) | 3.166 (2.915-3.438) | 8.249 (7.614-8.936) |
| **12** | **Amiodarone** | 0.793 (0.463-1.360) | 2.702 (0.565-12.917) | 2.449 (0.537-11.181) | 1.615 (0.355-7.357) | 1.475 (0.317-6.854) | 1.944 (0.440-8.582) |
|  | **Antiarythmics Ia** | 1.229 (0.713-2.121) | 3.030 (0.638-14.394) | 2.822 (0.626-12.732) | 1.513 (0.335-6.832) | 2.451 (0.531-11.326) | 6.592 (1.511-28.758) |
| **13** | **Methotrexate** | 1.053 (0.841-1.319) | 1.178 (0.881-1.575) | 1.212 (0.895-1.641) | 1.143 (0.811-1.611) | 3.327 (1.649-6.712) | 8.384 (4.233-16.604) |
|  | **Omeprazole** | 0.556 (0.454-0.682) | 0.976 (0.747-1.276) | 0.730 (0.552-0.965) | 0.384 (0.280-0.527) | 4.842 (2.430-9.650) | 13.207 (6.740-25.878) |
| **14** | **Simvastatin** | 1.665 (0.989-2.804) | 1.727 (0.630-4.732) | 1.578 (0.571-4.361) | 1.178 (0.397-3.499) | 1.013 (0.388-2.647) | 1.840 (0.742-4.562) |
|  | **Gemfibrozil** | 1.312 (0.768-2.239) | 2.147 (0.792-5.825) | 2.614 (0.960-7.121) | 2.013 (0.686-5.911) | 1.251 (0.481-3.253) | 2.307 (0.942-5.653) |
| **15** | **Simvastatin** | 0.970 (0.886-1.062) | 1.378 (1.147-1.657) | 1.119 (0.931-1.344) | 0.922 (0.761-1.118) | 3.099 (2.362-4.066) | 8.019 (6.159-10.442) |
|  | **Clarithromycin** | 1.326 (1.213-1.450) | 8.437 (7.032-10.123) | 11.194 (9.314-13.453) | 8.725 (7.197-10.578) | 4.796 (3.659-6.286) | 14.517 (11.134-18.926) |
| **16** | **Betablockers** | 0.782 (0.692-0.884) | 1.173 (0.936-1.470) | 1.562 (1.254-1.946) | 1.637 (1.306-2.050) | 4.163 (2.897-5.982) | 10.067 (7.065-14.345) |
|  | **Verapamil** | 0.870 (0.765-0.989) | 1.254 (0.986-1.595) | 0.915 (0.725-1.154) | 0.536 (0.423-0.679) | 4.620 (3.197-6.675) | 9.870 (6.885-14.148) |
| **17** | **Simvastatin** | 0.812 (0.710-0.927) | 1.402 (0.987-1.994) | 2.228 (1.593-3.118) | 2.966 (2.118-4.154) | 3.557 (2.308-5.482) | 6.149 (4.021-9.405) |
|  | **Verapamil** | 1.205 (1.051-1.383) | 2.005 (1.402-2.866) | 2.431 (1.730-3.415) | 1.884 (1.342-2.645) | 4.588 (2.974-7.079) | 8.035 (5.254-12.288) |
| **18** | **Enalapril** | 2.258 (2.112-2.414) | 2.061 (1.719-2.471) | 2.953 (2.482-3.514) | 3.909 (3.293-4.640) | 3.057 (2.598-3.598) | 6.244 (5.331-7.314) |
|  | **Allopurinol** | 1.049 (0.981-1.121) | 1.979 (1.653-2.369) | 2.684 (2.257-3.191) | 3.280 (2.763-3.893) | 2.312 (1.964-2.723) | 3.279 (2.796-3.845) |
| **19** | **Warfarin** | 0.991 (0.935-1.051) | 1.747 (1.511-2.021) | 2.145 (1.872-2.458) | 1.835 (1.604-2.099) | 4.045 (3.334-4.908) | 10.932 (9.042-13.218) |
|  | **NSAIDs or ASA** | 1.513 (1.445-1.584) | 2.335 (2.058-2.648) | 4.911 (4.362-5.528) | 5.661 (5.032-6.368) | 7.099 (5.899-8.541) | 27.584 (23.013-33.063) |
| **20** | **Methotrexate** | 0.743 (0.625-0.883) | 1.460 (1.181-1.804) | 1.656 (1.307-2.098) | 1.140 (0.877-1.481) | 4.070 (3.034-5.460) | 8.165 (6.087-10.953) |
|  | **NSAIDs or ASA** | 0.634 (0.573-0.700) | 0.864 (0.762-0.979) | 0.638 (0.559-0.728) | 0.331 (0.283-0.386) | 5.774 (4.535-7.351) | 19.029 (15.014-24.118) |
| **21** | **Enalapril** | 1.510 (1.447-1.577) | 2.146 (1.945-2.368) | 3.243 (2.950-3.565) | 4.170 (3.799-4.579) | 3.571 (3.278-3.890) | 6.312 (5.802-6.866) |
|  | **ASA** | 1.149 (1.109-1.190) | 1.778 (1.621-1.950) | 2.289 (2.095-2.500) | 2.674 (2.451-2.917) | 2.194 (2.022-2.380) | 3.214 (2.967-3.480) |
| **22** | **Enalapril** | 1.067 (0.998-1.142) | 2.207 (1.888-2.580) | 2.410 (2.070-2.806) | 1.461 (1.252-1.706) | 2.995 (2.564-3.497) | 5.922 (5.092-6.886) |
|  | **Metformin** | 1.076 (1.006-1.150) | 1.808 (1.549-2.112) | 2.561 (2.201-2.980) | 2.549 (2.184-2.974) | 2.496 (2.137-2.915) | 4.130 (3.550-4.804) |
| **23** | **Warfarin** | 0.754 (0.503-1.130) | 0.615 (0.323-1.170) | 0.255 (0.133-0.489) | 0.200 (0.105-0.381) | ND | ND |
|  | **Itraconazole** | 1.503 (1.000-2.258) | 2.842 (1.490-5.420) | 3.884 (1.999-7.547) | 5.449 (2.813-10.556) | ND | ND |
| **24** | **Warfarin** | 0.408 (0.361-0.462) | 1.638 (1.238-2.167) | 1.323 (1.012-1.730) | 0.965 (0.738-1.260) | 3.191 (2.101-4.846) | 6.114 (4.059-9.211) |
|  | **Levotiroxine** | 1.799 (1.588-2.038) | 2.752 (2.099-3.609) | 6.989 (5.383-9.074) | 11.257 (8.661-14.632) | 6.006 (3.973-9.078) | 17.126 (11.42-25.683) |
| **25** | **Simvastatin** | 1.081 (0.985-1.186) | 1.514 (1.110-2.065) | 2.663 (1.980-3.582) | 5.821 (4.342-7.804) | 7.923 (4.540-13.826) | 23.857 (13.764-41.352) |
|  | **Digoxin** | 1.250 (1.140- 1.372) | 2.944 (2.150- 4.032) | 2.941 (2.179- 3.971) | 1.652 (1.229- 2.219) | 6.986 (3.996- 12.214) | 16.195 (9.320- 28.142) |
| **26** | **ACE inhibitors** | 1.013 (0.995- 1.032) | 2.705 (2.622- 2.790) | 4.098 (3.973- 4.227) | 4.443 (4.307- 4.582) | 3.927 (3.826- 4.029) | 8.705 (8.474- 8.942) |
|  | **NSAIDs or ASA** | 1.190 (1.174- 1.206) | 3.589 (3.496- 3.684) | 5.818 (5.67- 5.971) | 7.418 (7.228- 7.612) | 3.122 (3.051- 3.195) | 6.073 (5.935- 6.215) |
| **27** | **SSRIs** | 0.884 (0.856- 0.913) | 2.040 (1.956- 2.127) | 3.237 (3.097- 3.384) | 3.566 (3.411- 3.727) | 3.961 (3.761- 4.172) | 9.943 (9.436- 10.477) |
|  | **NSAIDs or ASA** | 0.593 (0.579- 0.608) | 1.076 (1.038- 1.116) | 1.125 (1.085- 1.166) | 1.192 (1.151- 1.235) | 3.681 (3.507- 3.864) | 9.455 (9.013- 9.918) |

ND= not determinable (no cases of <5 drugs)

| **PANEL B** | | **Adjusted ORs (95% CI)** | | | | | |
| --- | --- | --- | --- | --- | --- | --- | --- |
|  |  | **SEX** | **AGE (years)** | | | **NUMBER of drugs** | |
|  |  | **male** | **50-64** | **65-74** | **≥75** | **5-9** | **≥10** |
| **1** | **Simvastatin** | 1.031 (0.746-1.425) | 1.085 (0.620-1.900) | 0.731 (0.412-1.297) | 0.435 (0.229-0.829) | 1.821 (0.829-4.003) | 5.166 (2.447-10.907) |
|  | **Itraconazole** | 1.814 (1.310-2.512) | 6.708 (3.815-11.796) | 9.828 (5.463-17.683) | 6.256 (3.239-12.084) | 3.416 (1.554-7.51) | 11.017 (5.162-23.513) |
| **2** | **Metformin** | 1.029 (0.963-1.100) | 1.660 (1.436-1.920) | 1.738 (1.505-2.007) | 1.754 (1.515-2.030) | 3.175 (2.647-3.809) | 7.581 (6.354-9.045) |
|  | **Fluoroquinolones** | 1.033 (0.969-1.101) | 4.567 (3.961-5.266) | 5.205 (4.516-5.999) | 4.081 (3.532-4.716) | 3.672 (3.065-4.4) | 9.036 (7.577-10.775) |
| **3** | **Omeprazole** | 3.455 (2.863-4.169) | 1.561 (1.205-2.021) | 0.946 (0.721-1.242) | 0.655 (0.492-0.873) | 9.624 (3.512-26.368) | 47.537 (17.672-127.878) |
|  | **Clopidogrel** | 1.286 (1.043-1.587) | 0.823 (0.613-1.107) | 0.570 (0.419-0.777) | 0.486 (0.350-0.675) | 5.316 (1.898-14.887) | 11.738 (4.263-32.322) |
| **4** | **Warfarin** | 1.401 (1.286-1.526) | 1.938 (1.562-2.405) | 1.835 (1.492-2.255) | 1.434 (1.167-1.762) | 3.591 (2.685-4.802) | 5.940 (4.462-7.906) |
|  | **Amiodarone** | 1.094 (1.000-1.197) | 2.238 (1.785-2.807) | 1.964 (1.583-2.437) | 1.146 (0.925-1.421) | 3.703 (2.748-4.989) | 4.492 (3.349-6.026) |
| **5** | **Warfarin** | 1.364 (0.973-1.912) | 1.389 (0.553-3.492) | 1.427 (0.597-3.410) | 1.556 (0.659-3.672) | 3.436 (0.801-14.727) | 8.313 (2.001-34.541) |
|  | **Moxifloxacin** | 1.393 (0.994-1.951) | 3.212 (1.282-8.048) | 5.985 (2.498-14.339) | 7.990 (3.367-18.963) | 5.468 (1.281-23.33) | 13.870 (3.339-57.614) |
| **6** | **Simvastatin** | 1.932 (1.704-2.191) | 1.784 (1.227-2.594) | 2.583 (1.797-3.713) | 4.115 (2.866-5.908) | 4.292 (2.433-7.571) | 14.578 (8.373-25.380) |
|  | **Amiodarone** | 1.280 (1.125-1.457) | 2.433 (1.654-3.579) | 2.096 (1.443-3.044) | 1.543 (1.065-2.235) | 3.481 (1.958-6.187) | 7.511 (4.277-13.192) |
| **7** | **Warfarin** | 1.280 (1.148-1.426) | 2.376 (1.762-3.204) | 2.299 (1.723-3.068) | 1.613 (1.207-2.154) | 2.666 (1.858-3.826) | 4.975 (3.498-7.077) |
|  | **Simvastatin** | 1.535 (1.379-1.708) | 1.736 (1.293-2.331) | 2.680 (2.016-3.562) | 3.278 (2.461-4.367) | 3.160 (2.210-4.519) | 7.109 (5.015-10.077) |
| **8** | **Digoxin** | 0.723 (0.642-0.814) | 2.234 (1.560-3.197) | 2.481 (1.777-3.464) | 1.967 (1.425-2.715) | 2.810 (1.942-4.065) | 3.358 (2.334-4.831) |
|  | **Verapamil** | 0.880 (0.778-0.996) | 1.451 (1.012-2.079) | 2.121 (1.520-2.959) | 4.150 (3.008-5.727) | 4.329 (2.994-6.260) | 7.002 (4.879-10.049) |
| **9** | **Warfarin** | 0.628 (0.550-0.718) | 1.166 (0.832-1.634) | 1.404 (1.028-1.916) | 1.397 (1.028-1.898) | 1.921 (1.289-2.865) | 3.517 (2.386-5.182) |
|  | **SSRIs** | 1.424 (1.248-1.625) | 2.685 (1.930-3.735) | 7.318 (5.395-9.927) | 9.142 (6.766-12.354) | 3.197 (2.156-4.74) | 7.304 (4.982-10.71) |
| **10** | **Verapamil** | 0.753 (0.476-1.191) | 1.164 (0.550-2.461) | 0.648 (0.305-1.376) | 0.350 (0.159-0.768) | 3.820 (1.124-12.987) | 8.015 (2.437-26.359) |
|  | **Atenolol** | 0.788 (0.499-1.245) | 0.972 (0.466-2.026) | 1.031 (0.493-2.155) | 1.177 (0.542-2.554) | 3.772 (1.119-12.716) | 11.027 (3.406-35.707) |
| **11** | **Eparines** | 1.007 (0.957-1.060) | 1.649 (1.486-1.830) | 2.205 (1.997-2.435) | 2.543 (2.310-2.799) | 2.742 (2.436-3.088) | 4.482 (3.988-5.037) |
|  | **Nimesulide, Indomethacin, or Acetylsalicylic acid** | 0.711 (0.677-0.747) | 1.163 (1.052-1.285) | 1.651 (1.503-1.813) | 2.390 (2.184-2.616) | 2.481 (2.212-2.783) | 5.695 (5.096-6.363) |
| **12** | **Amiodarone** | 1.151 (0.415-3.191) | 4.324 (0.467-40.003) | 1.739 (0.174-17.419) | 0.873 (0.083-9.229) | 0.426 (0.067-2.717) | 0.676 (0.132-3.467) |
|  | **Antiarythmics Ia** | 1.688 (0.605-4.707) | 4.657 (0.518-41.898) | 1.958 (0.202-18.985) | 0.783 (0.076-8.056) | 0.741 (0.117-4.683) | 2.385 (0.473-12.012) |
| **13** | **Methotrexate** | 1.060 (0.816-1.376) | 1.155 (0.822-1.622) | 1.278 (0.900-1.814) | 1.086 (0.724-1.629) | 3.068 (1.385-6.792) | 6.705 (3.092-14.541) |
|  | **Omeprazole** | 0.562 (0.441-0.718) | 0.969 (0.702-1.337) | 0.776 (0.556-1.083) | 0.373 (0.254-0.55) | 4.542 (2.073-9.952) | 11.302 (5.256-24.302) |
| **14** | **Simvastatin** | 2.891 (1.133-7.375) | 1.115 (0.275-4.526) | 0.955 (0.226-4.039) | 1.142 (0.256-5.097) | 0.959 (0.237-3.873) | 1.326 (0.341-5.156) |
|  | **Gemfibrozil** | 2.255 (0.871-5.842) | 1.351 (0.341-5.348) | 1.556 (0.380-6.367) | 1.974 (0.456-8.541) | 1.192 (0.302-4.710) | 1.681 (0.450-6.282) |
| **15** | **Simvastatin** | 0.840 (0.722-0.977) | 1.600 (1.178-2.173) | 1.088 (0.798-1.484) | 0.830 (0.598-1.152) | 2.813 (1.823-4.341) | 6.876 (4.515-10.471) |
|  | **Clarithromycin** | 1.168 (1.005-1.356) | 9.762 (7.182-13.268) | 10.864 (7.936-14.873) | 7.891 (5.663-10.995) | 4.353 (2.822-6.713) | 12.613 (8.254-19.276) |
| **16** | **Betablockers** | 0.760 (0.611-0.945) | 1.173 (0.792-1.738) | 1.483 (1.009-2.179) | 1.671 (1.128-2.476) | 4.479 (2.397-8.371) | 8.91 (4.818-16.476) |
|  | **Verapamil** | 0.847 (0.679-1.058) | 1.241 (0.829-1.857) | 0.875 (0.59-1.298) | 0.567 (0.381-0.845) | 4.788 (2.550-8.992) | 8.02 (4.315-14.904) |
| **17** | **Simvastatin** | 0.837 (0.721-0.972) | 1.444 (0.975-2.138) | 2.181 (1.497-3.177) | 3.147 (2.160-4.583) | 3.660 (2.276-5.886) | 5.572 (3.489-8.898) |
|  | **Verapamil** | 1.238 (1.063-1.443) | 2.057 (1.382-3.060) | 2.347 (1.606-3.430) | 1.981 (1.358-2.889) | 4.713 (2.928-7.586) | 7.216 (4.521-11.518) |
| **18** | **Enalapril** | 2.288 (2.112-2.478) | 2.272 (1.819-2.837) | 3.138 (2.533-3.888) | 4.169 (3.375-5.149) | 2.997 (2.478-3.624) | 5.23 (4.346-6.293) |
|  | **Allopurinol** | 1.092 (1.008-1.183) | 2.179 (1.746-2.718) | 2.885 (2.329-3.573) | 3.587 (2.902-4.432) | 2.275 (1.880-2.754) | 2.826 (2.345-3.407) |
| **19** | **Warfarin** | 0.878 (0.810-0.951) | 1.406 (1.139-1.736) | 1.755 (1.442-2.136) | 1.467 (1.207-1.784) | 3.077 (2.316-4.088) | 6.328 (4.792-8.358) |
|  | **NSAIDs or ASA** | 1.347 (1.251-1.452) | 2.063 (1.690-2.519) | 4.622 (3.839-5.566) | 5.016 (4.169-6.036) | 5.940 (4.501-7.841) | 22.022 (16.795-28.875) |
| **20** | **Methotrexate** | 0.754 (0.641-0.886) | 1.313 (1.075-1.605) | 1.255 (1.010-1.560) | 0.937 (0.732-1.199) | 3.330 (2.446-4.534) | 4.920 (3.629-6.670) |
|  | **NSAIDs or ASA** | 0.613 (0.547-0.687) | 0.862 (0.750-0.992) | 0.606 (0.522-0.704) | 0.305 (0.255-0.364) | 5.596 (4.289-7.301) | 17.800 (13.710-23.112) |
| **21** | **Enalapril** | 1.149 (1.109-1.190) | 1.778 (1.621-1.950) | 2.289 (2.095-2.500) | 2.674 (2.451-2.917) | 2.194 (2.022-2.380) | 3.214 (2.967-3.480) |
|  | **ASA** | 1.172 (1.126-1.219) | 1.779 (1.604-1.973) | 2.329 (2.110-2.571) | 2.743 (2.489-3.023) | 2.074 (1.900-2.263) | 2.679 (2.458-2.919) |
| **22** | **Enalapril** | 1.040 (0.964-1.121) | 2.295 (1.921-2.742) | 2.523 (2.121-3.000) | 1.562 (1.310-1.864) | 2.976 (2.507-3.532) | 5.267 (4.456-6.226) |
|  | **Metformin** | 1.043 (0.968-1.124) | 1.905 (1.596-2.273) | 2.702 (2.272-3.212) | 2.708 (2.270-3.230) | 2.481 (2.090-2.946) | 3.702 (3.130-4.378) |
| **23** | **Warfarin** | 1.015 (0.505-2.038) | 0.405 (0.144-1.137) | 0.180 (0.063-0.510) | 0.151 (0.054-0.417) | ND | ND |
|  | **Itraconazole** | 2.087 (1.034-4.213) | 1.825 (0.645-5.165) | 2.594 (0.891-7.555) | 3.840 (1.337-11.029) | ND | ND |
| **24** | **Warfarin** | 0.378 (0.324-0.442) | 2.140 (1.473-3.110) | 1.787 (1.246-2.563) | 1.305 (0.910-1.870) | 2.533 (1.583-4.052) | 4.189 (2.645-6.633) |
|  | **Levotiroxine** | 1.604 (1.373-1.875) | 3.553 (2.463-5.125) | 9.301 (6.522-13.265) | 14.886 (10.425-21.257) | 4.889 (3.070-7.784) | 12.031 (7.630-18.972) |
| **25** | **Simvastatin** | 1.088 (0.977-1.212) | 1.997 (1.328-3.003) | 3.519 (2.374-5.215) | 7.929 (5.370-11.707) | 8.412 (4.317-16.394) | 22.769 (11.769-44.052) |
|  | **Digoxin** | 1.257 (1.129-1.400) | 3.863 (2.559-5.832) | 3.891 (2.617-5.787) | 2.309 (1.561-3.416) | 7.283 (3.731-14.219) | 15.255 (7.867-29.582) |
| **26** | **ACE inhibitors** | 1.031 (1.013-1.050) | 2.446 (2.363-2.531) | 3.521 (3.404-3.642) | 3.955 (3.825-4.090) | 3.375 (3.280-3.474) | 5.832 (5.666-6.002) |
|  | **NSAIDs or ASA** | 1.182 (1.164-1.200) | 3.574 (3.465-3.687) | 5.805 (5.631-5.984) | 7.471 (7.247-7.701) | 3.060 (2.979-3.144) | 5.552 (5.406-5.702) |
| **27** | **SSRIs** | 0.857 (0.826-0.890) | 2.055 (1.948-2.167) | 3.105 (2.942-3.276) | 3.478 (3.298-3.668) | 3.354 (3.133-3.591) | 6.558 (6.130-7.016) |
|  | **NSAIDs or ASA** | 0.578 (0.560-0.597) | 1.199 (1.141-1.260) | 1.343 (1.279-1.411) | 1.476 (1.407-1.549) | 3.459 (3.239-3.694) | 8.675 (8.134-9.252) |

ND= not determinable (no cases of <5 drugs)
